# COVID-19 severity scale for claims data research

**DOI:** 10.1186/s12913-023-09362-2

**Published:** 2023-04-26

**Authors:** Trudy Millard Krause, Raymond Greenberg, Lopita Ghosh, Joseph S. Wozny, Regina M. Hansen, Caroline Schaefer

**Affiliations:** 1 Department of Management, Policy and Community Health, School of Public Health, The University of Texas Health Science Center at Houston, Houston, United States; 2 Department of Population and Data Sciences, University of Texas Southwestern Medical Center, Dallas, United States

**Keywords:** COVID-19, Disease Severity, Administrative Claims Healthcare, Healthcare cost, Epidemiological methods, SARS-CoV-2

## Abstract

**Objective:**

To create and validate a methodology to assign a severity level to an episode of COVID-19 for retrospective analysis in claims data.

**Data Source:**

Secondary data obtained by license agreement from Optum provided claims records nationally for 19,761,754 persons, of which, 692,094 persons had COVID-19 in 2020.

**Study Design:**

The World Health Organization (WHO) COVID-19 Progression Scale was used as a model to identify endpoints as measures of episode severity within claims data. Endpoints used included symptoms, respiratory status, progression to levels of treatment and mortality.

**Data Collection/Extraction methods:**

The strategy for identification of cases relied upon the February 2020 guidance from the Centers for Disease Control and Prevention (CDC).

**Principal Findings:**

A total of 709,846 persons (3.6%) met the criteria for one of the nine severity levels based on diagnosis codes with 692,094 having confirmatory diagnoses. The rates for each level varied considerably by age groups, with the older age groups reaching higher severity levels at a higher rate. Mean and median costs increased as severity level increased. Statistical validation of the severity scales revealed that the rates for each level varied considerably by age group, with the older ages reaching higher severity levels (p < 0.001). Other demographic factors such as race and ethnicity, geographic region, and comorbidity count had statistically significant associations with severity level of COVID-19.

**Conclusion:**

A standardized severity scale for use with claims data will allow researchers to evaluate episodes so that analyses can be conducted on the processes of intervention, effectiveness, efficiencies, costs and outcomes related to COVID-19.

**Supplementary Information:**

The online version contains supplementary material available at 10.1186/s12913-023-09362-2.

## Highlights

A novel COVID-19 severity scale is developed for researchers who use claims data.

Costs increase with severity of COVID-19 episodes.

Demographic factors such as age, race, geographic region, and comorbidity count had statistically significant associations with severity level of COVID-19.

**Supplementary Information:**

The online version contains supplementary material available at 10.1186/s12913-023-09362-2.

## Introduction

COVID-19, the illness caused by Severe Acute Respiratory Syndrome (SARS) coronavirus 2, arose as a global pandemic in 2020, continuing into 2021. Symptoms of the disease range widely from none to death, and result in high demand on health care providers, hospitals and resources, such as emergency care services and inpatient beds, intensive care beds and complex life-saving equipment. Correspondingly, the median costs associated with COVID-19 treated cases were estimated by Bartsch in 2020 to range from $3,994 for mild symptomatology to $18,579 for hospitalized cases [1]. A later study by Tsai et al. used Medicare claims revealing higher costs for hospitalization, at a mean of $21,752 up to $49,441 if mechanical ventilation was required [2]. Several studies have shown that patient characteristics such as age, gender, and comorbidities have impacted both the risk for infection as well as the severity of illness [3, 4, 5].

The World Health Organization (WHO), working with the International Forum for Acute Care Trialists and the International Severe Acute Respiratory and Emerging Infections Consortium, developed a WHO Clinical Progression Scale to measure the viral burden of COVID-19 and to assess the patient trajectory and resources used over the course of COVID-19 [6]. Other scoring instruments have been developed for emergency department triage or prediction of mortality [7]. These tools are primarily utilized for identification of patient risk to optimize inpatient management of patients.

To date no known tool exists to assign a severity level to an episode of COVID-19 for purposes other than patient management. We propose a reliable and reproducible method to allow researchers to evaluate episodes as opposed to acute hospitalization so that analyses can be conducted on the processes of treatment intervention, treatment effectiveness, treatment efficiencies, and health care costs and outcomes related to COVID-19.

Healthcare claims data are available through payor sources such as health insurance carriers, Medicare and Medicaid, as well as from companies that aggregate, de-identify and license use of large administrative claims databases. Health claims data provide valuable information on insured persons across time regardless of provider or provider group. Specific diagnoses and procedures are documented for services and exist in the data both historically and linked to treatment events by date. Thus, claims data allow for identification of specific individuals or cohorts who meet designated medical or demographic criteria, allowing the researcher to evaluate co-morbidities, utilization patterns, costs of services, define episodes of care and measure outcomes both individually and population based. Claims data research has been used effectively for policy analyses and to inform population health initiatives.

Claims data are, however, subject to a time lag, whereby the provider submission of a claim for reimbursement follows the actual service, and the adjudication of the claim by the payor is a process that also delays data. Typically, claims data are added to the database when the claim is processed and paid by the carrier, so often are incomplete until 90 days after submission. As the COVID-19 pandemic began in the United States in early 2020 and continues to date, the full year of 2020 claims were determined to be available in April 2021 for analysis.

## Methods

The database used for the development of this scale was Optum’s Clinformatics® Data Mart (CDM) which is derived from administrative health claims for members of large commercial and Medicare Advantage health plans (Optum® de-identified COVID-19 Electronic Health Record dataset (2007–2020). The database includes approximately 19 million annual covered lives, for a total of over 65 million unique lives over a 9-year period (1/2007 through 12/2020). Clinformatics® Data Mart is statistically de-identified under the Expert Determination method consistent with HIPAA and managed according to Optum® customer data use agreements. CDM administrative claims submitted for payment by providers and pharmacies are verified, adjudicated and de-identified prior to inclusion. These data, including patient-level enrollment information, are derived from claims submitted for all medical and pharmacy health care services with information related to health care costs and resource utilization. The population is geographically diverse, spanning all 50 states.

## Results

The COVID-19 scale was applied to the national claims data in the Optum CDM for all medical claims in 2020. Of the 19,761,754 total unique persons with enrollment information in the database in 2020, 692,094 (3.5%) met the criteria for one of the severity levels based on diagnosis codes. The age distribution was as expected with infection rates generally rising with increasing age (Table 1).

**Table 1:** Percentage of persons in each age group with a COVID-19 diagnosis

Age (years)	Persons	Persons with a COVID DX	Percent
0–19	3,047,218	44,380	1.46%
20–34	3,501,120	122,581	3.50%
25–44	2,345,512	83,550	3.56%
45–54	2,238,285	90,034	4.02%
55–64	2,323,533	94,035	4.05%
65–74	3,521,560	121,348	3.45%
75 +	2,784,526	136,166	4.89%
Total	19,761,754	692,094	3.50%

As shown in Table 2, over half of all patients –(60%), fell into Severity Level 2 – a confirmed diagnosis of COVID-19 but asymptomatic and ambulatory. Another 14% remained ambulatory (Level 3), resulting in 72% of diagnosed cases that did not require a higher level of care. 12% utilized the emergency department (Level 4) but did not require admission, and the remaining 12% (Levels 5–9) were hospitalized at various levels of severity. Slightly more than 2% died during hospitalization (Level 9). The rates for each level varied considerably by age group, with the older ages reaching higher severity levels (p < 0.001). Other demographic factors such as race and ethnicity, geographic region, and comorbidity count had statistically significant associations with severity level of COVID-19 (Table 2).

**Table 2:** Distribution of severity by age, gender, race, region, comorbidities and cost

	Severity Level n								
	Level 2 416,766 (60.4%)	Level 3 96,798 (14.0%)	Level 4 81,038 (11.7%)	Level 5 23,236 (3.4%)	Level 6 20,687 (3%)	Level 7 32,488 (4.7%)	Level 8 2,818 (0.4%)	Level 9 16,711 (2.4%)	
**Age Group n, (%)**									*
0–19	35,038 (79.22)	5650 (12.77)	3275 (7.4)	141 (0.32)	70 (0.16)	50 (0.11)	3 (0.01)	1 (0)	
20–34	88,693 (72.53)	16,848 (13.78)	14,471 (11.83)	865 (0.71)	775 (0.63)	574 (0.47)	29 (0.02)	27 (0.02)	
25–44	56,011 (67.19)	13,191 (15.82)	11,142 (13.37)	928 (1.11)	787 (0.94)	1142 (1.37)	92 (0.11)	73 (0.09)	
45–54	56,696 (63.12)	14,682 (16.34)	12,380 (13.78)	1597 (1.78)	1427 (1.59)	2557 (2.85)	224 (0.25)	264 (0.29)	
55–64	55,152 (58.78)	14,437 (15.39)	12,408 (13.22)	2878 (3.07)	2501 (2.67)	4831 (5.15)	502 (0.53)	1126 (1.20)	
65–74	62,990 (52)	16,305 (13.46)	14,686 (12.12)	6219 (5.13)	5507 (4.55)	9921 (8.19)	977 (0.81)	4532 (3.74)	
75 +	62,186 (45.77)	15,685 (11.54)	12,676 (9.33)	10,608 (7.81)	9620 (7.08)	13,413 (9.87)	991 (0.73)	10,688 (7.87)	
**Gender**									*
Female	229,464 (61.5)	52,159 (13.98)	43,865 (11.76)	12,145 (3.25)	11,207 (3.00)	15,744 (4.22)	1205 (0.32)	7334 (1.97)	
Male	187,280 (59.01)	44,631 (14.06)	37,169 (11.71)	11,091 (3.49)	9478 (2.99)	16,743 (5.28)	1613 (0.51)	9376 (2.95)	
**Race** ^**+**^									*
Unknown	51,159 (63.25)	11,214 (13.86)	9491 (11.73)	2230 (2.76)	1992 (2.46)	2962 (3.66)	236 (0.29)	1598 (1.98)	
Asian	12,578 (65.36)	2658 (13.81)	1738 (9.03)	653 (3.39)	434 (2.26)	691 (3.59)	71 (0.37)	422 (2.19)	
Black	38,214 (50.48)	9546 (12.61)	11,182 (14.77)	3834 (5.07)	3627 (4.79)	5484 (7.24)	841 (1.11)	2966 (3.92)	
Hispanic	62,687 (56.22)	15,581 (13.97)	17,389 (15.6)	3982 (3.57)	3266 (2.93)	4953 (4.44)	573 (0.51)	3070 (2.75)	
White	252,128 (62.53)	57,799 (14.33)	41,238 (10.23)	12,537 (3.11)	11,368 (2.82)	18,398 (4.56)	1097 (0.27)	8655 (2.15)	
**Region**									*
Midwest	106,096 (60.2)	26,715 (15.16)	20,273 (11.5)	5573 (3.16)	5075 (2.88)	8576 (4.87)	515 (0.29)	3427 (1.94)	
Northeast	64,486 (68.3)	11,509 (12.19)	5052 (5.35)	4926 (5.22)	2182 (2.31)	2658 (2.82)	301 (0.32)	3297 (3.49)	
South	174,492 (56.34)	44,483 (14.36)	44,427 (14.34)	9056 (2.92)	10,963 (3.54)	16,846 (5.44)	1628 (0.53)	7831 (2.53)	
West	71,692 (65.08)	14,091 (12.79)	11,286 (10.25)	3681 (3.34)	2467 (2.24)	4408 (4.00)	374 (0.34)	2156 (1.96)	
**Comorbid†**									*
None	213,022 (73.83)	41,552 (14.4)	29,475 (10.22)	1757 (0.61)	1347 (0.47)	1119 (0.39)	13 (0)	249 (0.09)	
1 or more	203,744 (50.68)	55,246 (13.74)	51,563 (12.83)	21,479 (5.34)	19,340 (4.81)	31,369 (7.80)	2805 (0.70)	16,462 (4.09)	

Percent represents the proportion of a severity level within the designated category of the covariable.

^*^Represents p-value < 0.05 of Chi-square test for independence.

^+^Race is self-reported by enrollee. “White” and “Black” does not imply “Non-Hispanic White” or “Non-Hispanic Black” as the designation is not an option.

^†^Comorbidities include: diabetes w/o complications, diabetes w/ complications, hypertension w/o complications, hypertension w/ complications, ischemic heart disease, CKD, asthma, COPD, rheumatoid arthritis, dementia, obesity.

Costs also varied significantly by severity level, which was an expected finding as the levels were related to intensity of resource use which drive costs. Table 3 presents the mean and median costs per person in relation to severity level. Patients who reached severity level 8 and survived incurred the highest average costs related to COVID-19 at a median of $197,007. The highest severity level 9 had lower median costs than the level below, which is likely explained by the death in hospital resulting in shorter lengths of stay. In Table 4, the gamma regression analysis shows how each predictor increases compared to the reference level while holding all other variables constant. The regression shows a significant relationship between cost and severity, in that more severe cases predicted higher costs while controlling for the other factors. The most pronounced difference can be found between severity levels 3 and 4. Severity level 4 is exp(1.86): 6.42 times the cost of level 3. Similar trends were observed in higher levels such as 6.05 times the cost in level 5 compared to level 4, and 2.53 times the cost in level 8 compared to level 7.

**Table 3:** Mean and Median cost of care by severity level for patients with COVID-19

	Total Charges	Median Charges Per Person	Mean Charges Per Person	Median Length of Stay	Mean Length of Stay
Severity level 1	N/A	N/A	N/A	NA	NA
Severity level 2	1,459,113,499	$147	$297	NA	NA
Severity level 3	1,658,932,188	$222	$641	NA	NA
Severity level 4	4,813,304,025	$2,604	$3,680	NA	NA
Severity level 5	1,071,136,975	$43,440	$62,617	7	13
Severity level 6	3,532,831,095	$53,587	$79,944	6	10
Severity level 7	1,459,113,499	$77,800	$147,769	8	12
Severity level 8	1,658,932,188	$197,007	$379,297	12	18
Severity level 9	4,813,304,025	$131,499	$210,864	9	12

NA = Not Applicable because level did not have designated charges or did not require hospitalization.

**Table 4:** Generalized Gamma Regression of Cost and Severity

Parameter	Estimate	95% Confidence Limits		Wald ChiSq	p-value
Intercept	9.51	9.50	9.52	2200937.00	< 0.0001
Level 3 vs. 2	0.78	0.78	0.79	43494.40	< 0.0001
Level 4 vs. 3	1.86	1.85	1.87	143336.00	< 0.0001
Level 5 vs. 4	1.80	1.79	1.82	53771.10	< 0.0001
Level 6 vs. 5	0.25	0.23	0.27	660.26	< 0.0001
Level 7 vs. 6	0.61	0.60	0.63	4472.12	< 0.0001
Level 8 vs. 7	0.93	0.89	0.97	2120.26	< 0.0001
Level 9 vs. 8	-0.64	-0.68	-0.60	935.56	< 0.0001
20–34	0.08	0.07	0.09	210.92	< 0.0001
25–44	0.13	0.11	0.14	420.62	< 0.0001
45–54	0.15	0.14	0.16	570.41	< 0.0001
55–64	0.31	0.30	0.33	2470.36	< 0.0001
65–74	0.34	0.33	0.36	2940.91	< 0.0001
75+	0.47	0.46	0.48	5451.64	< 0.0001
Midwest	-0.17	-0.18	-0.17	2995.03	< 0.0001
Northeast	0.10	0.09	0.10	600.06	< 0.0001
West	-0.03	-0.03	-0.02	46.50	< 0.0001
Unknown Race	0.12	0.11	0.13	888.78	< 0.0001
Asian	0.11	0.10	0.13	224.91	< 0.0001
Black	0.14	0.13	0.15	1089.88	< 0.0001
Hispanic	0.13	0.12	0.13	1272.14	< 0.0001
Female	-0.06	-0.07	-0.06	628.92	< 0.0001
Comorbidities > = 1	0.45	0.45	0.46	18626.90	< 0.0001
Scale	0.94	0.94	0.95		

Levels refer to severity levels. White is baseline for race, south for regions and men for gender, Cost is continuous outcome variable.

## Discussion

This study relied on claims data to identify persons with confirmed COVID-19. Confirmation of COVID-19 was determined by a diagnosis code on a claim submitted by a provider for medical or pharmacy services. The prevalence of confirmed COVID-19 cases at 3.6% was lower in the claims data than generally reported for the population. Sen et al. reported that 33% of the US population had been infected by the end of 2020 yet only 11.8% were documented, providing a comparative estimate of 3.9% of the population with documented infection [8]. The lower rate may be due to several factors including (1) testing not recorded with a health care claim, (2) diagnosis not assigned on testing claim, (3) cases confirmed through non-billed sources, such as public health agencies resulting in undercounting due to care received without a related bill for service [9], (4) selection bias in studying only persons with commercial health insurance [9].

The COVID-19 pandemic impacted the health care system through excessive demands on resources such as intensive care facilities, over-taxed capacity of hospitals, and shortage of health care providers which may have influenced the progression of an individual’s disease severity. These factors could not be controlled for in the model for evaluation. An additional limitation was the issue related to high cost claimants whose claims were allocated to a stop-loss account once an annual limit was reached and subsequent charges were not reported. This may have resulted in lower observed costs than actual costs incurred, however, the number of affected individuals was small and any resulting bias in cost estimates likely was minor.

The recorded average costs are consistent with the studies by Bartsch and Tsai, with ambulatory patients incurring costs less than $3,000 and increasing for hospitalized cases with wide variation in overall mean of $21,752 to $47,441 for Medicare patients [1, 2]. What has not been demonstrated previously is the extent to which health care costs are driven by the most severely affected patients. As the present index was created based on the intensity of medical interventions, which are directly related to costs. What was not appreciated prior to this evaluation was the exponential shape of the cost curve, with the small number of patients receiving the most intensive care driving overall costs.

We believe that the COVID-19 scale would be useful for further research on both clinical and financial impacts of the disease. Severity Level 1 has limited utility for a cost analysis because it represented a small number of patients with limited information. However, the authors believe that this level may have potential value in other contexts, such as the exploration of long-term outcomes (i.e. “post-acute COVID”) and its impact on comorbid conditions.

The authors present this scale for application by researchers who use claims data to evaluate the impact of the COVID-19 pandemic on individuals, populations, and on policy. Standardization of a measure of severity would allow easier comparison of results across studies and facilitate a determination of reproducibility of findings in various settings and populations. The scale can be implemented in any claims-based dataset such as those maintained by health plans, researchers, and health systems with claims based records. It is relevant across age groups, sex, and payor groups (i.e. Medicare, Medicaid, commercial, etc.). Future COVID-19 research will likely include analyses of the severity of COVID-19 events and the impact on continuing symptoms or complications. Additionally, over time, the value of Level 1 may increase as COVID-19 cases are less frequently documented by a provider and self-report increases. Finally, in the future the use of the severity scale may benefit from the addition of information on vaccination history for which various codes have been created.

To build the logic for the claims-based COVID-19 Severity Scale (referred to as the COVID-19 Scale) we modeled it upon the design of the WHO Progression Scale. For hospitalized patients, this scale relied on clinical values documented in medical records, with a special focus on oxygen levels (FiO_2_ and pO_2_) and use of mechanical ventilation, renal dialysis and extracorporeal membrane oxygenation (ECMO) as key measures for patients at the highest levels of severity [6]. From data measured over the course of treatment, the WHO scale identified 10 levels of patient progression as follows: Score 0: Uninfected, Scores 1, 2, and 3: Ambulatory mild disease, Scores 4 or 5: hospitalized: moderate disease, Scores 6,7,8,or 9: Hospitalized: severe diseases and Score 10: Dead.

Since documentation of oxygen levels (FiO2 and p02) is not available in claims data, we modeled similar endpoints as used in the WHO scale, using information that is routinely available within retrospective claims data. The modified measure is intended to be used as an index of episode severity as opposed to treatment progression. Endpoints commonly used included symptoms, respiratory status, progression to levels of treatment (ambulatory, emergency department, inpatient admission), and mortality. For patients who required oxygen therapy we relied upon documentation of the use of various levels of respiratory treatments, with specific focus on mechanical ventilation. Renal dialysis and ECMO are well documented in claims data and were incorporated for patients who required these additional treatments. Death is not always well documented in claims data, unless it occurs in an inpatient setting, for which discharge status is coded as “expired.”

The strategy for identification of cases between January 1, 2020 and April 2020 before an official ICD-10 diagnosis code was issued for COVID-19 relied upon the February 2020 guidance from the Centers for Disease Control and Prevention (CDC) that combined codes for respiratory infections with code B97.29 – other coronavirus [10]. Another challenge was evident in that many persons during the pandemic developed a presumptive COVID-19 infection without a confirming diagnosis or a medical claim submitted by a provider for detection or treatment. Additionally, individuals began to experience sequelae of COVID-19 without an earlier diagnosis in the claims data. For these cases, the CDC published additional guidance with ICD-10 codes for “personal history of COVID-19” or “sequelae of COVID-19” [11]. The publication of the ICD-10 code for COVID-19 in April 2020 allowed confirmed cases to be documented in claims data. The coding logic is detailed in Appendix A and includes the ICD-10 codes used in the COVID-19 Scale.

The original intent of the scale was to identify persons who had COVID-19 at any time during 2020, and to assign the highest severity level experienced by each person. If a person had more than one documented episode of COVID-19, the highest severity level was assigned to that person so that person-based research would capture the most debilitating state attained.

Like the WHO Progression Scale, we tiered the COVID-19 Scale by ordinal levels according to symptomatology, resource use, and mortality. The individual ranks are clearly defined, mutually exclusive, and ordered in a hierarchical progression reflecting clinical` deterioration [11]. Levels 1–3 had no documentation of presentation to or treatment at an emergency department or an inpatient facility yet are differentiated by documentation of diagnosis and symptoms (Level 1: no documented diagnosis but personal report of COVID-19, Level 2: diagnosis code but no symptom code, Level 3: diagnosis code and symptom code(s)). Because the scale was used initially to assess “subacute COVID” and other individual health impacts, we defined Level 1 to represent a personal history of COVID as documented in the claims without confirmatory diagnostic evidence. Because less than 1% met criteria for Level 1 - no confirmatory diagnosis yet personal report of COVID-19, this level was excluded from further analysis because a diagnosis did not exist in the data.

The other ambulatory-only levels were delineated by the existence of symptoms Levels 2 and 3. Level 4 reflected treatment for COVID-19 at an emergency department without inpatient admission. Levels 5–9 all required an inpatient admission with progressively increasing levels of resource use or procedures reflecting respiratory distress or organ failure (Level 5: Hospital admit no oxygen, Level 6: Hospital admit with non-invasive oxygen, Level 7: Hospital admit with mechanical ventilation, Level 8: Hospital admit with mechanical ventilation and renal dialysis or ECMO). Level 9 indicated death during the hospital treatment. See Appendix B for detailed Level definitions.

Costs were computed for each patient with COVID-19 in 2020, considering only the costs associated with claims that included a COVID-19 diagnosis. Total costs were based upon both hospital/facility and professional charges. It was noted that approximately 1% of the claims in the database had the charges and paid amounts recorded as “$0” or $0.01”, and it was determined that these were claims that exceeded an annual individual stop-loss amount for that member. In these cases, the commercial health plan reallocated the claim to a stop-loss policy and the excess amount is shown as “0” but all other claim details remain in the data. These cases were excluded from the cost analysis because the total cost could not be computed.

A generalized gamma regression analysis was used because the univariate relationship between severity level and cost was non-linear. Severity levels were backwards difference coded to compare each level to the level directly prior in the regression. A sensitivity analysis was done on only those identified as having COVID-19 after April 2020 when the COVID-19 ICD-10 code was developed. Results were near identical to original regression with complete population, verifying the measures (Appendix C). This analysis was performed using SAS software, Version 9.4 of the SAS System [12]. This study was reviewed and approved by the Institutional Review Board of the University of Texas Health Science Center at Houston, and all methods were performed in accordance with the relevant guidelines and regulations.

## Electronic supplementary material

Below is the link to the electronic supplementary material.

Supplementary Material 1

## Data Availability

The database used for the development of this scale was Optum’s Clinformatics® Data Mart (CDM) which is derived from administrative health claims for members of large commercial and Medicare Advantage health plans. The data that support the findings of this study are available from Optum but restrictions apply to the availability of these data, which were used under license for the current study, and so are not publicly available. Aggregated data findings may however beavailable from the authors upon reasonable request and with permission of Optum.

## References

[1] Bartsch SM, Ferguson MC, McKinnell JA, O’Shea KJ, Wedlock PT, Siegmund SS, Lee BY. The Potential Health Care Costs And Resource Use Associated With COVID-19 In The United States. Health Affairs, 10.1377/hlthaff.2020.00426, HEALTH AFFAIRS 39, NO. 6 (2020): 927–935 10.1377/hlthaff.2020.00426 PMC11027994 32324428

[2] Tsai Y, Vogt TM, Zhou F. Patient Characteristics and Costs Associated With COVID-19–Related Medical Care Among Medicare Fee-for-Service Beneficiaries,Ann Intern Med. doi:10.7326/M21-1102 10.7326/M21-1102 PMC8252832 34058109

[3] Miethke-Morais A, Cassenote A, Piva H (2021). COVID-19-related hospital cost-outcome analysis: the impact of clinical and demographic factors. Braz J Infect Dis.

[4] Huespe I, Carboni Bisso I, Di Stefano S, Terrasa S, Gemelli NA, Las Heras M (2020).

[5] Altschul DJ, Unda SR, Benton J (2020). A novel severity score to predict inpatient mortality in COVID-19 patients. Sci Rep.

[6] WHO Working Group on the Clinical Characterisation and Management of COVID-19 infection. A minimal common outcome measure set for COVID-19 clinical research. Lancet Infect Dis. 2020 Aug;20(8):e192-e197. doi: 10.1016/S1473-3099(20)30483-7. Epub 2020 Jun 12. Erratum in: Lancet Infect Dis. 2020 Oct;20(10):e250. PMID: 32539990; PMCID: PMC7292605. 10.1016/S1473-3099(20)30483-7 PMC7292605 32539990

[7] Haimovich AD, Ravindra NG, Stoytchev S, van Dijk D, Schulz WL, Taylor RA. Development and Validation of the Quick COVID-19 Severity Index: A Prognostic Tool for Early Clinical Decompensation.Infectious Disease/Original Research, Vol 76, Issue 4, p442–453, October 01, 2020 10.1016/j.annemergmed.2020.07.022 PMC7373004 33012378

[8] Sen P, Yamuna TK, Candela S et al. Burden and characteristics of COVID-19 in the United States during 2020. Nature 598, 338–341 (2021). 10.1038/s41586-021-03914-4 Accessed October 26, 2021 10.1038/s41586-021-03914-4 34438440

[9] Majumder MS, Rose S, Health Care Claims Data May Be Useful For COVID-19 Research Despite Significant Limitations. " Health Affairs Blog October. 2020;6. 10.1377/hblog20201001.9773.

[10] Centers for Disease Control CDC., ICD-10-CM Official Coding Guidelines – Supplement, Coding encounters related to COVID-19 Coronavirus Outbreak, Effective: February 20, 2020COVID-10 clinical presentation: https://www.cdc.gov/coronavirus/2019-nCoV/hcp/clinical-criteria.html

[11] Centers for Disease Control CDC, ICD-10-CM Official Guidelines for Coding and, Reporting. FY 2021 – UPDATED January 1, 2021, (October 1, 2020 - September 30, 2021) ICD-10-CM Official Guidelines for Coding and Reporting, FY 2021, https://www.cms.gov/files/document/2021-coding-guidelines-updated-12162020.pdf

[12] SAS Institute Inc (2013). SAS/ACCESS® 9.4 interface to ADABAS: reference.

