# Supplementary material for: COVID-19 severity scale for claims data research
Source: BMC Health Serv Res. 2023 Apr 26;23:402. doi: 10.1186/s12913-023-09362-2 (PMC10131339; doi:10.1186/s12913-023-09362-2)
Supplement: Supplementary file 1 — Supplementary Material 1 [file 12913_2023_9362_MOESM1_ESM.docx]

Appendix A: Coding Logic for 2020 COVID-19

| THE FOLLOWING CODES ARE CONFIRMATION OF COVID 19 DIAGNOSES | |
| --- | --- |
| U07.1 | COVID-19, |
| U07.2 | Clinically-epidemiologically diagnosed COVID-19 |
| U10 | Multisystem inflammatory syndrome (MIS) associated with COVID 19 |
| THE FOLLOWING CODES ARE TIME LIMITED TO JAN-APRIL 2020 BEFORE THE U CODES WERE ISSUED AND ARE CONSIDERED EARLY CONFIRMATION OF COVID | |
| J12.82 AND B97.29 DATED BETWEEN 1/1/2020-4/30/2020 | For a pneumonia case confirmed as due to the 2019 novel coronavirus (COVID-19), assign codes J12.89, Other viral pneumonia, and B97.29, Other coronavirus as the cause of diseases classified elsewhere. |
| J20.8 AND B97.29 DATED BETWEEN 1/1/2020-4/30/2020 | For a patient with acute bronchitis confirmed as due to COVID-19, assign codes J20.8, Acute bronchitis due to other specified organisms, and B97.29, Other coronavirus as the cause of diseases classified elsewhere |
| J40x AND B97.29 DATED BETWEEN 1/1/2020-4/30/2020 | Bronchitis not otherwise specified (NOS) due to the COVID-19 should be coded using code J40, Bronchitis, not specified as acute or chronic; along with code B97.29, Other coronavirus as the cause of diseases classified elsewhere. |
| J22x AND B97.29 DATED BETWEEN 1/1/2020-4/30/2020 | If the COVID-19 is documented as being associated with a lower respiratory infection, not otherwise specified (NOS), or an acute respiratory infection, NOS, this should be assigned with code J22, Unspecified acute lower respiratory infection, with code B97.29 |
| J98.8AND B97.29 DATED BETWEEN 1/1/2020-4/30/2020 | Other coronavirus as the cause of diseases classified elsewhere. If the COVID-19 is documented as being associated with a respiratory infection, NOS, it would be appropriate to assign code J98.8, Other specified respiratory disorders, with code B97.29, Other coronavirus as the cause of diseases classified elsewhere. |
| J80x AND B97.29 DATED BETWEEN 1/1/2020-4/30/2020 | Cases with ARDS due to COVID-19 should be assigned the codes J80, Acute respiratory distress syndrome, and B97.29, Other coronavirus as the cause of diseases classified elsewhere. |
| THE FOLLOWING CODES ARE PRESUMPTIVE OF COVID IF THERE IS NO OTHER COVID DIAGNOSIS CODE | |
| U08 in 2020 or later | personal history of COVID-19 |
| Z86.16 in 2020 or later | personal history of COVID-19 |
| THE FOLLOWING CODES IDENTIFY POST COVID CONDITIONS AND SEQUELAE AND MAY APPEAR WITHOUT A CONFIRMATORY COVID CODE PRIOR | |
| U09 WITH any other dx code | Post COVID 19 condition |
| B94.8 with history of COVID 19 dx | Sequelae of COVID-19 B94.8, Sequelae of other specified infectious and parasitic diseases. Use this code for patients who are still suffering from COVID-19 complications after clearing the initial infection. It should be combined with other diagnosis codes for the patient’s ongoing symptoms. |

Appendix B: Definition of COVID-19 Severity Scale

| Level | Definition | Coding Logic |
| --- | --- | --- |
| 1 | No confirmatory evidence yet personal history of Covid 19 recorded AND NO PROGRESSION TO A HIGHER LEVEL OF SEVERITY | Only COVID-19 Code that exists is Z86.16 or U08 OR U09 or B94.8 *Reasoning: the codes noted represent a personal history code, which would be used for self-report or otherwise unconfirmed/undocumented clinical diagnosis* |
| 2 | Outpatient or professional claim contains a code that indicates COVID 19 but no further level of treatment and **asymptomatic**: claim has COVID DX code U07.1 or U07.2 or U10 or if occurring Jan 2020-April 2020 the following combinations: (J12.82 AND B97.29) or (J20.8 AND B97.29) or (J40x AND B97.29) or (J22x AND B97.29) or (J98.8 AND B97.29) or (J80x AND B97.29) AND ON THE SAME CLAIM NOT any dx code representing a symptom and no progression to a higher level of severity | *Reasoning: this would be the group with documented COVID-19 diagnosis and asymptomatic, but the person did not progress to use of an ED or inpatient admission, therefore considered outpatient/ambulatory only and low severity* |
| 3 | Outpatient or professional claim contains a code that indicates COVID 19 but no further level of treatment and **SYMPTOMATIC**: claim has COVID DX code U07.1 or U07.2 or U10 or if occurring Jan 2020-April 2020 the following combinations: (J12.82 AND B97.29) or (J20.8 AND B97.29) or (J40x AND B97.29) or (J22x AND B97.29) or (J98.8 AND B97.29) or (J80x AND B97.29) AND ON THE SAME CLAIM any dx code indicating symptoms and no progression to a higher level of severity | *Reasoning: this would be the group with documented COVID-19 diagnosis and symptomatic, but the person did not progress to use of an ED or inpatient admission, therefore considered outpatient/ambulatory only and low severity*  *For Level 3 there must also be documentation of pneumonia, or acute bronchitis or lower respiratory infection or acute respiratory distress syndrome, or cough, or shortness of breath, or fever which are identified by the CDC as symptoms of COVID 19: https://www.cdc.gov/nchs/data/icd/COVID-19-guidelines-final.pdf* |
| 4 | Emergency department treatment as shown by ED revenue code on claim with a COVID-19 diagnosis and no subsequent admission | ED Revenue code: 450-459 or 0450-0459 *Reasoning: these persons progressed beyond only outpatient/ambulatory care to a need to present to the emergency department, yet did not progress further to admission or confirmed death* |
| 5 | Hospital admission with a COVID 19 diagnosis code and no indication of a higher level of treatment, no use of oxygen | *Reasoning: these persons had a hospital admission for COVID, but did not progress to the high levels of inpatient treatment/services as indicated in the following stages* |
| 6 | Hospital admission with a COVID 19 diagnosis code with use of non-invasive oxygen, and no indication of a higher level of treatment | CPT codes for non-invasive oxygen: 94660 (CPAP, BiPAP), 94662 (CNP) , 94779 (unlisted pulmonary) OR REV code 0270 , 0175, 0998, 0272 |
| 7 | Hospital admission with a COVID 19 diagnosis code and mechanical ventilation during the hospitalization | LOGIC: search for Proc code: CPT 4 = 94002, 94003, 94004, 94005 or ICD 10 PCS code = 5A1955Z or 5A1935Z or 5A1945Z or 5A093(any) 5A094(any) or 5A095(any) OR CPT CODE 31500 OR REV CODE 0410 *Reasoning: these persons were hospitalized and received mechanical ventilation indicating respiratory issues* |
| 8 | Hospital admission with a COVID 19 diagnosis code, mechanical ventilation, and renal dialysis OR ECMO | Renal dialysis codes: Revenue codes 800-809 or 0800-0809, or ICD PCD codes: 5A1D00Z or 5A1D60Z or 3E1M39Z *Reasoning: these persons required renal dialysis when treated for COVID-19 indicating organ failure ECMO: CPT Codes 33946-33959, 33962-33966, 33969, 33984-33989 OR ICD 10 PCS Codes: 5A1522F, 5A1522G, 5A1522H Reasoning: these persons required ECMO when treated for COVID-19 indicating severe cardiac issues* |
| 9 | Hospital stay with a COVID 19 diagnosis code, AND discharge status of expired or death | *Reasoning: these persons were discharged from a COVID-19 hospitalization (or SNF or Rehab) as "expired" indicating death during the admission episode* |

**Appendix C: Sensitivity Analysis**

Title: Generalized Gamma Regression of Cost and Severity in those with COVID-19 Diagnosis after April 2020

| **Parameter** | **Estimate** | **95% Confidence Limits** | | **Wald ChiSq** | **p-value** |
| --- | --- | --- | --- | --- | --- |
| **Intercept** | **9.51** | **9.50** | **9.52** | **2195624.00** | **<.0001** |
| **Level 3 vs 2** | **0.79** | **0.78** | **0.79** | **43553.40** | **<.0001** |
| **Level 4 vs 3** | **1.86** | **1.85** | **1.87** | **142371.00** | **<.0001** |
| **Level 5 vs 4** | **1.80** | **1.79** | **1.82** | **53535.60** | **<.0001** |
| **Level 6 vs 5** | **0.25** | **0.23** | **0.27** | **660.13** | **<.0001** |
| **Level 7 vs 6** | **0.61** | **0.60** | **0.63** | **4461.57** | **<.0001** |
| **Level 8 vs 7** | **0.93** | **0.89** | **0.97** | **2114.00** | **<.0001** |
| **Level 9 vs 8** | **-0.64** | **-0.68** | **-0.60** | **920.13** | **<.0001** |
| **20-34** | **0.08** | **0.07** | **0.09** | **208.97** | **<.0001** |
| **25-44** | **0.13** | **0.11** | **0.14** | **420.76** | **<.0001** |
| **45-54** | **0.15** | **0.14** | **0.16** | **569.58** | **<.0001** |
| **55-64** | **0.31** | **0.30** | **0.33** | **2459.51** | **<.0001** |
| **65-74** | **0.34** | **0.33** | **0.35** | **2930.10** | **<.0001** |
| **75+** | **0.47** | **0.46** | **0.48** | **5443.55** | **<.0001** |
| **Midwest** | **-0.17** | **-0.18** | **-0.17** | **2990.72** | **<.0001** |
| **Northeast** | **0.10** | **0.09** | **0.11** | **623.89** | **<.0001** |
| **West** | **-0.02** | **-0.03** | **-0.02** | **44.55** | **<.0001** |
| **Unknown** | **0.12** | **0.11** | **0.13** | **889.96** | **<.0001** |
| **Asian** | **0.11** | **0.10** | **0.13** | **213.81** | **<.0001** |
| **Black** | **0.14** | **0.13** | **0.15** | **1094.65** | **<.0001** |
| **Hispanic** | **0.13** | **0.12** | **0.13** | **1261.52** | **<.0001** |
| **Female** | **-0.06** | **-0.07** | **-0.06** | **631.08** | **<.0001** |
| **Comorbidities >=1** | **0.45** | **0.45** | **0.46** | **18597.40** | **<.0001** |
| **Scale** | **0.94** | **0.94** | **0.95** |  |  |
